# Autochthonous Case of *Rickettsia slovaca* Infection in Russia

**DOI:** 10.3201/eid2710.204621

**Published:** 2021-10

**Authors:** Ruslan F. Sayfullin, Nadezhda E. Perekopskaya, Ludmila S. Karan, Nadezhda N. Zvereva, Muhammad A. Sayfullin

**Affiliations:** Municipal Clinical Hospital No 52, Moscow, Russia (R.F. Sayfullin);; Pirogov Russian National Research Medical University, Moscow (R.F. Sayfullin, N.N. Zvereva, M.A. Sayfullin);; Infectious Clinical Hospital No. 1, Moscow (N.E. Perekopskaya);; Central Scientific Research Institute of Epidemiology, Moscow (L.S. Karan);; Gamaleya Institute of Epidemiology and Microbiology, Moscow (M.A. Sayfullin).

**Keywords:** bacteria, rickettsial infections, Rickettsia slovaca, spotted fever group rickettsiosis, tick-borne infections, ticks, Russia, vector-borne infections

## Abstract

We describe an autochthonous case of *Rickettsia slovaca* infection in a man 35 years of age from Russia who had tickborne lymphadenopathy. We used ELISA and quantitative PCR testing to further identify DNA and confirm diagnosis. Physicians in Russia should consider similar diseases in differential diagnoses after tick bites.

*Rickettsia slovaca* was isolated in *Dermacentor marginatus* ticks in 1968 in Slovakia and recognized as a *Rickettsia* species with unknown pathogenicity. In 1997, a study described the first laboratory-confirmed case of *Rickettsia slovaca* infection in a human (*1*). *R. slovaca* has been detected in ticks in many countries in Europe, including the Mediterranean region. Human cases of syndromes that can be caused by *R. slovaca*, including tickborne lymphadenopathy (TIBOLA), *Dermacentor*-borne necrosis-erythema-lymphadenopathy (DEBONEL), and scalp eschar and neck lymphadenopathy after tick bite (SENLAT) have been reported (*2*,*3*). *R. slovaca* has been detected in ticks in 4 of 85 regions of Russia (Figure), and 1 imported case of *R. slovaca* infection was reported (*4*–*7*). The aim of our study was to describe an autochthonous case of *R. slovaca* infection in a man in Russia.

**Figure:**
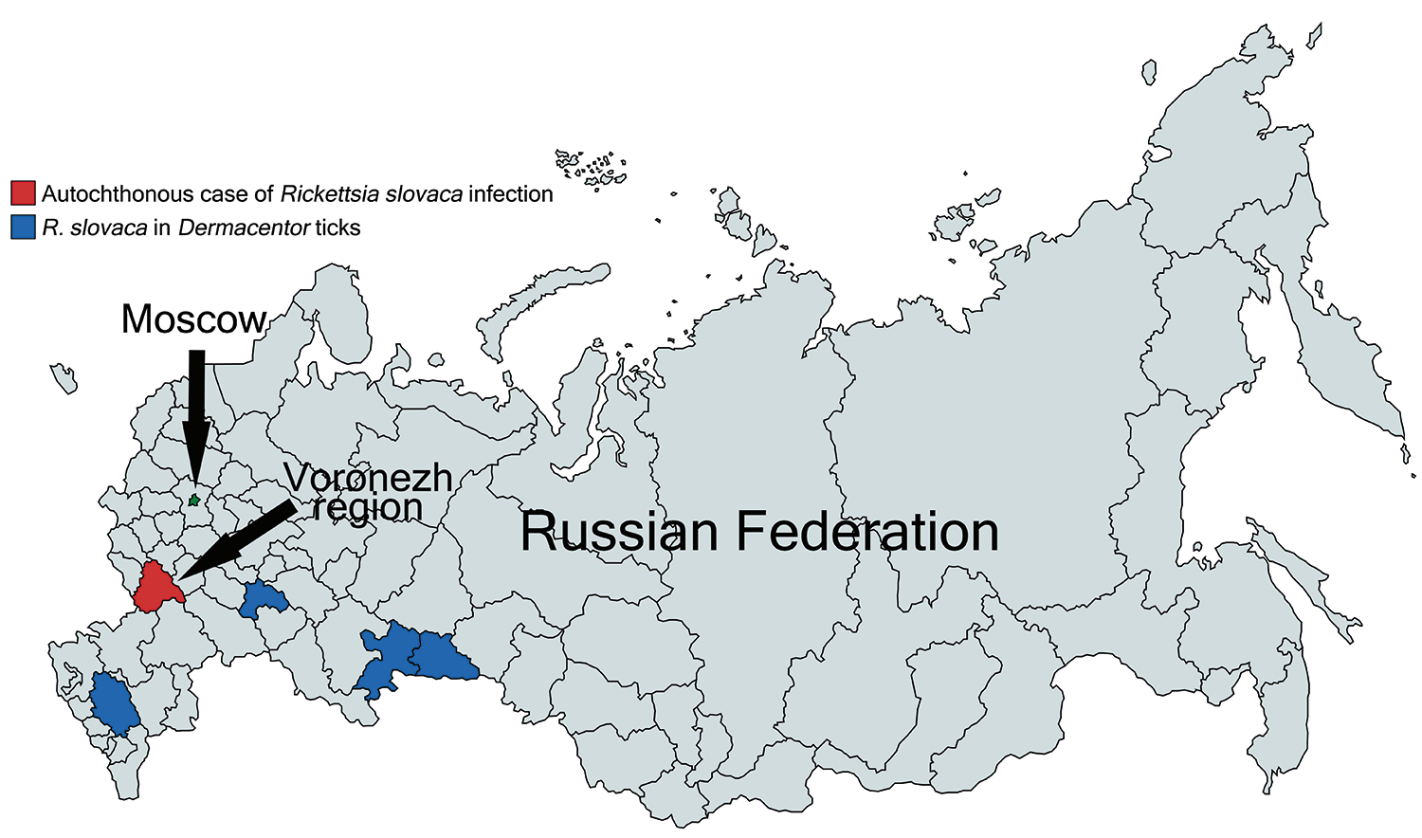
Regions in Russia where *Rickettsia slovaca* was detected only in ticks and the region where an autochthonous human case of *R. slovaca* infection was registered.

In May 2019, a 35-year-old male resident of Russia with an unremarkable medical history sought treatment for eschar on the skin of his right shin, painful and enlarged inguinal lymph nodes, rash, pain in his right knee, and severe fatigue. Before onset, he was in rural village in the Voronezh region of Russia for 8 days, where he had contact with domestic animals and later noticed an insect bite near the location of the eschar. He reported no history of foreign travel in the previous 6 months.

Disease onset began with an ulcer, 2–3 cm in diameter, on his right shin. By days 3–4, the ulcer became an eschar, and the patient experienced chills and sweats at night. By days 5–6, chills and sweats remained, and an erythema up to 5 cm in diameter appeared around the eschar. The patient also noticed pain in his right knee, papular rash on his right leg and the right side of his trunk and neck, and enlarged and painful inguinal and axillary lymph nodes. On day 6, he was examined by a surgeon, who suspected a skin infection and initiated amoxicillin (1.5 g/d). On days 7–8, the rash spread to other limbs, lymph nodes in his neck became painful and enlarged, the pain in his right knee worsened, and low-grade fever (37.4°C–37.6°C) developed.

On day 8 after symptom onset, he was hospitalized at Infectious Clinical Hospital No. 1 in Moscow. At admission, he had a black eschar surrounded by erythema on the upper part of his right shin and vesiculopapular rash on his limbs and trunk concentrated around the eschar and on the skin of the right knee; in addition, there was bright hyperemia of previously existing scratches. Inguinal, axillary, and neck lymph nodes were painful by palpation and enlarged to 1.5–2.0 cm; his right knee was enlarged and painful by palpation and had impaired range of motion. We found no abnormalities from complete blood count and urinalysis on admission. We suspected skin and soft tissue infection with knee arthritis and changed antimicrobial therapy to ceftriaxone (2.0 g/d) and metronidazole (1.5 g/d). 

On day 10, his body temperature normalized and the erythema around the eschar faded, but the rash continued to spread (Appendix) and the pain in his knee worsened. He had slightly elevated C-reactive protein (12 mg/L; reference <5 mg/L), but urine and blood cultures showed no growths. Taking into account anamnesis and a black eschar typical of TIBOLA, DEBONEL, and SENLAT syndromes, we suspected rickettsiosis. We detected *Rickettsia* DNA, but only in the sample from the eschar swab sample. We confirmed *R. slovaca* infection by molecular assay on blood and the eschar swab samples, collected on day 10 after disease onset (Appendix). We extracted DNA using a QIAGEN DNeasy blood and tissue kit (https://www.qiagen.com) and tested it with an AmpliSens *Rickettsia* spp. SFG-FL real-time PCR kit (https://www.amplisens.ru). For further confirmation, we used DNA isolated from the swab to sequence partial OmpA (primers Rr190.70p, Rr190.701n) and gltA (primers RpCS.877p, RpCS.1258n) genes (*8*). 

We changed the patient’s antimicrobial therapy to doxycycline (0.2 g/d), and his health improved rapidly. By day 15, pain and edema in his right knee had regressed, the rash had faded, and the eschar had begun to heal. The patient was discharged, but continued taking doxycycline for 10 additional days.

By 2 months after disease onset, the eschar and a few elements of papular rash around it had completely disappeared, but substantial fatigue remained for up to 5 months. For serologic assays, we collected serum samples on days 10, 30, and 160 after disease onset and tested for *Rickettsia* IgM and IgG using a Vircell *Rickettsia conorii* ELISA IgG/IgM kit (https://www.vircell.com). The lack of serologic response that we observed may have been related to the sensitivity of the ELISA test we used (*9*). On the basis of our findings, physicians should consider TIBOLA, DEBONEL, and SENLAT syndromes in differential diagnoses after tick bites occurring in Russia. 

Appendix Materials and methods used to confirm *Rickettsia slovaca* infection in a patient in Russia.
