# Supplementary material for: Autochthonous Case of Rickettsia slovaca Infection in Russia
Source: Emerg Infect Dis. 2021 Oct;27(10):2736–8. doi: 10.3201/eid2710.204621 (PMC8462319; doi:10.3201/eid2710.204621)
Supplement: Appendix — Materials and methods used to confirm Rickettsia slovaca infection in a patient in Russia. [file 20-4621-Techapp-s1.pdf]

Article DOI: https://doi.org/10.3201/eid2710.204621

Autochthonous Case of *Rickettsia slovaca* Infection in Russia

Appendix

Supplementary Methods

For molecular assay we used samples of EDTA blood (the buffy coat fraction) and the eschar swab samples, all samples was collected on day 10 after disease onset. We collected dry sterile swab from the inoculation eschar as described elsewhere. (*1*). Before swabbing, we removed a scab from the eschar. The swab was directed toward the base of the eschar at a 50°–60° angle, while being rotated 5–6 times then placed back in the tube and immediately transported to the laboratory. DNA was extracted using a Qiagen DNeasy blood and tissue kit (https://www.qiagen.com) and tested at the Central Research Institute of Epidemiology, Moscow, Russia using an AmpliSens *Rickettsia* spp. real-time PCR kit SFG-FL (https://www.amplisens.ru) according to the manufacturer's instructions and using the internal, negative, and positive controls. The PCR was performed in a Rotor Gene Q real-time PCR cycler (Qiagen).

To identify *Rickettsia* species, we used DNA isolated from the eschar swab to sequence partial OmpA (primers Rr190.70p, Rr190.701n) and gltA (primers RpCS.877p, RpCS.1258n) genes (*2*). The purified PCR products were sequenced bidirectionally using BigDye Terminator v1.1 Cycle Sequencing kit (Thermo Fisher Scientific, https://www.thermofisher.com) on an Applied Biosystems3500xL Genetic Analyzer (Applied Biosystems, https://www.biosciences.ie). The obtained sequences were deposited in the NCBI GenBank under the accession numbers MT511329 and MT511330. Serum samples for serology assay were collected on days 10, 30, and 160 after the onset of disease and tested for Rickettsia IgM and IgG using the *Rickettsia conorii* ELISA IgG/IgM kit (https://www.vircell.com) according to the manufacturer’s instructions.

References

1. Mouffok N, Socolovschi C, Renvoise A, Parola P, Raoult D. Diagnosis of rickettsioses from eschar swab samples, Algeria. Emerg Infect Dis. 2011;17:1968–9. PubMed https://doi.org/10.3201/eid1710.110332

2. Portillo A, de Sousa R, Santibáñez S, Duarte A, Edouard S, Fonseca IP, et al. Guidelines for the detection of *Rickettsia* spp. Vector Borne Zoonotic Dis. 2017;17:23–32. PubMed https://doi.org/10.1089/vbz.2016.1966

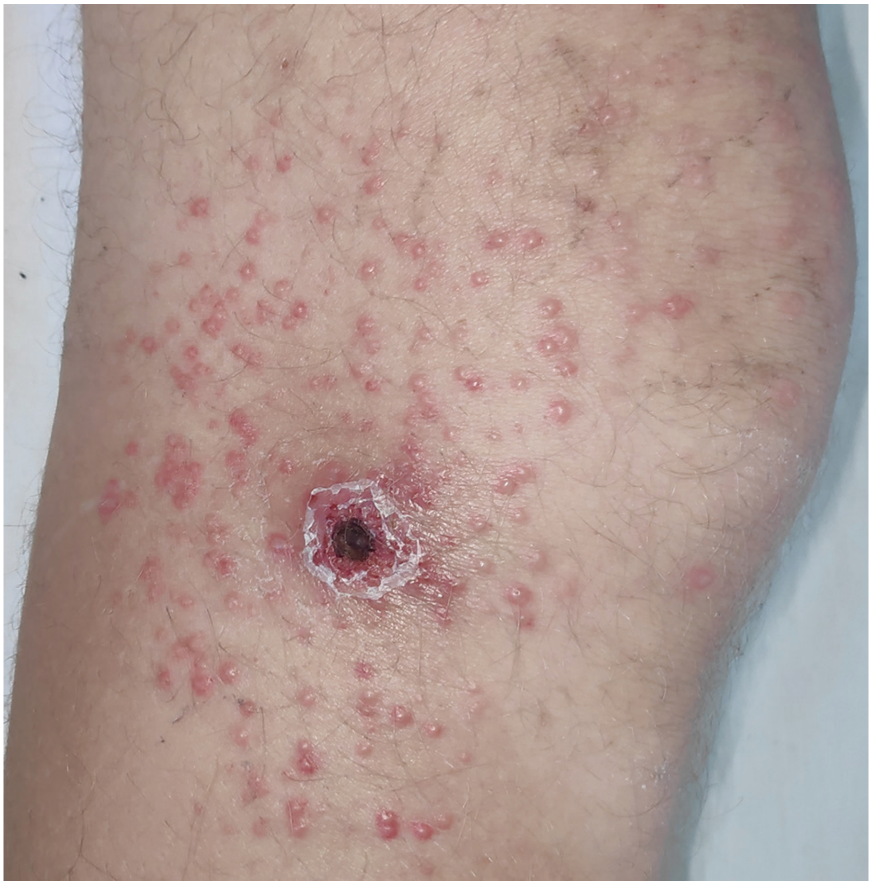

Appendix Figure. The patient's eschar, surrounded by a vesicular-papular rash on day 10 after disease onset, before initiating doxycycline.
